# VERSE: a novel approach to detect virus integration in host genomes through reference genome customization

**DOI:** 10.1186/s13073-015-0126-6

**Published:** 2015-01-20

**Authors:** Qingguo Wang, Peilin Jia, Zhongming Zhao

**Affiliations:** Department of Biomedical Informatics, Vanderbilt University School of Medicine, Nashville, TN 37203 USA; Center for Quantitative Sciences, Vanderbilt University Medical Center, Nashville, TN 37232 USA; Department of Psychiatry, Vanderbilt University School of Medicine, Nashville, TN 37232 USA; Department of Cancer Biology, Vanderbilt University School of Medicine, Nashville, TN 37232 USA

## Abstract

Fueled by widespread applications of high-throughput next generation sequencing (NGS) technologies and urgent need to counter threats of pathogenic viruses, large-scale studies were conducted recently to investigate virus integration in host genomes (for example, human tumor genomes) that may cause carcinogenesis or other diseases. A limiting factor in these studies, however, is rapid virus evolution and resulting polymorphisms, which prevent reads from aligning readily to commonly used virus reference genomes, and, accordingly, make virus integration sites difficult to detect. Another confounding factor is host genomic instability as a result of virus insertions. To tackle these challenges and improve our capability to identify cryptic virus-host fusions, we present a new approach that detects Virus intEgration sites through iterative Reference SEquence customization (VERSE). To the best of our knowledge, VERSE is the first approach to improve detection through customizing reference genomes. Using 19 human tumors and cancer cell lines as test data, we demonstrated that VERSE substantially enhanced the sensitivity of virus integration site detection. VERSE is implemented in the open source package VirusFinder 2 that is available at http://bioinfo.mc.vanderbilt.edu/VirusFinder/.

**Electronic supplementary material:**

The online version of this article (doi:10.1186/s13073-015-0126-6) contains supplementary material, which is available to authorized users.

## Background

Pathogenic viruses pose significant threats to public health throughout the world [1,2]. With rapid advances in next generation sequencing (NGS) technologies over the past several years and their potential to unbiasedly and comprehensively identify pathogens in clinical samples [3-6], numerous studies were conducted recently to shed light on disease-associated viruses, such as hepatitis B virus (HBV), human immunodeficiency virus (HIV), and human papillomavirus (HPV). One notable development, which has greatly enhanced our knowledge of the molecular mechanisms of viruses in tumor development, is the use of NGS to survey virus integration in cancer genomes among large cohorts of cancer patients [7-9] as well as their effects on host cell gene expression [10,11].

Virus insertions in host genomes typically cause host genomic instability [7-9], which is often evidenced by elevated adjacent mutation rates. The surrounding mutations (and the homology between the viral and host genomes) make the alignment of short reads to the host reference genomes difficult and, consequently, the detection of virus integration sites challenging. Another factor that adversely impacts the detection is viral sequence divergence as a result of high virus mutation rates [12,13], which makes the NGS reads sampled from the real virus genomes less likely to align to the commonly used virus reference sequences. The rapid genetic changes of the virus sequences require customized (or personalized) virus references that can take into account virus polymorphisms and evolution.

Stimulated by the strong demand for NGS investigations of virus-host interactions, a large number of tools were developed in the past three years for virus/virome characterization [14-20], novel infectious agent detection [14,21], and virus mutation spectrum analysis [22-25]. Along with progress in computational technologies and improvements in the NGS technologies, software designed specifically to detect virus integration in host genomes has also emerged, for example, VirusSeq [26], ViralFusionSeq [27], VirusFinder [28], and Virana [29]. However, these tools align NGS reads directly to the known virus and host reference genomes and, hence, cannot tackle effectively the challenges posed by virus-induced host genomic instability and viral genome variability. To improve our capability to identify cryptic virus-host fusions, novel approaches are still urgently required.

To facilitate the rapidly growing number of studies on disease-associated viruses, here we present a new approach that detects Virus intEgration sites through Reference SEquence customization (VERSE). The rationale of VERSE is to use short reads to iteratively 'correct' reference genomes so as to create new 'personalized' reference genomes. Corrections made to the references improve read mapability, and, accordingly, detection sensitivity. VERSE is specifically designed with the diversity and scale of today’s NGS applications and computational efficiency in mind. It allows quick analysis of NGS data of various types: whole genome sequencing (WGS), whole transcriptome sequencing (RNA-seq), targeted sequencing (TS), and so on. VERSE is implemented in a publicly available software package, VirusFinder [30]. In its sensitive detection mode (see the user’s manual at [30]), VirusFinder runs VERSE to characterize virus integration loci.

## Methods

### Next generation sequencing data

We used WGS of 13 hepatocellular carcinomas (HCCs), RNA-seq of 4 HCC cell lines, and TS of 2 Merkel cell carcinomas to evaluate VERSE (Table 1). All these samples are publicly available and were validated to harbor virus integration sites.

**Table 1:** **Human tumor samples that harbor validated virus integration sites**

**Tumor type**	**Virus**	**Number of samples**	**Sequencing technology**	**Number of integration sites**	**Accession number**	**Reference**
Hepatocellular carcinoma	HBV	13	WGS	20^a^	ERP001196	[7]
Hepatocellular carcinoma	HBV	4	RNA-seq	11	SRP023539	[11]
Merkel cell carcinoma	MCV	2	TS	3	NA	[31]

^a^Two virus insertion loci were considered as one if the genomic distance between them was less than 10 bp. HBV, hepatitis B virus; MCV, Merkel cell polyomavirus; NA, no accession number is associated with the project; RNA-seq, whole transcriptome sequencing; TS, targeted sequencing; WGS, whole genome sequencing.

Paired-end WGS (2 × 90 bp) of the 13 HCCs was performed on an Illumina HiSeq 2000 sequencer as described in [7]. Average coverage of these samples ranged from 31.7× to 121.2× (Table S1 in Additional file 1). The HBV integration sites identified in these samples were validated using PCR and Sanger resequencing [7]. In total, 22 integration events were validated in these tumor samples. Several samples harbored virus integration sites that were very close to each other. For example, the two HBV insertion sites in sample 145 T, chr19: 30303492 and chr19: 30303498, were only 6 bp away (Table S1 in Additional file 1). The discrimination of virus integration sites within this short distance is quite beyond the capability of current detection tools. Because VERSE applies a 10 bp cutoff to filter out low-confidence detections, we regarded two virus insertion loci as one if the distance between them was less than 10 bp. This gave us a final set of 20 virus integration sites for these samples.

Whole transcriptomes of the four HCC cell lines were subjected to sequencing library preparation using an Illumina TruSeq RNA Sample Preparation Kit as reported in the original publication [11]. Sequencing was performed on an Illumina HiSeq 2000 platform, generating paired-end reads of length 101 bp with an average insertion size of 300 bp (Table S2 in Additional file 1). On average, 127 million reads were obtained per sample. Eleven chimeric HBV-human transcripts were detected in these samples using ViralFusionSeq [27] and validated using Sanger resequencing.

For the two Merkel cell carcinomas, virus genomes were captured from formalin-fixed, paraffin-embedded tissues and enriched using PCR-generated capture probes [31]. Targeted paired-end sequencing was performed on an Illumina GAIIx platform (2 × 100 bp). In total, 3.9 and 5.0 million reads were produced for the two samples, respectively. The viral integration sites in the tumor genomes were detected using both BreakDancer [32] and SLOPE [33]. To validate the identified virus insertion events, primers were designed using Vector NTI suite (Invitrogen). For a detailed validation protocol, interested readers are referred to [31].

Besides the data from the real tumors and cancer cell lines, we also simulated WGS of human chromosome 1 using the profile-based Illumina pair-end Read Simulator (pIRS) [34]. We plugged a mutated copy of the HPV-16 virus reference genome (GI:310698439) into chromosome 1 of UCSC hg19 to create a new reference with which to run the command '*simulate'* in pIRS to generate paired-end sequencing reads (insert size: 200 bp; read length: 2 × 75 bp; average coverage: 30×). Additionally, to mimic real data, we inserted single nucleotide polymorphisms (SNPs), small insertions and deletions (indels), and structural variants (SVs) into the data. We let the frequency of SNPs be 10 times higher than that of indels and the frequency of SVs 10 times less than that of indels. The simulation data are freely available at [30].

### VERSE pipeline

**Figure 1:**
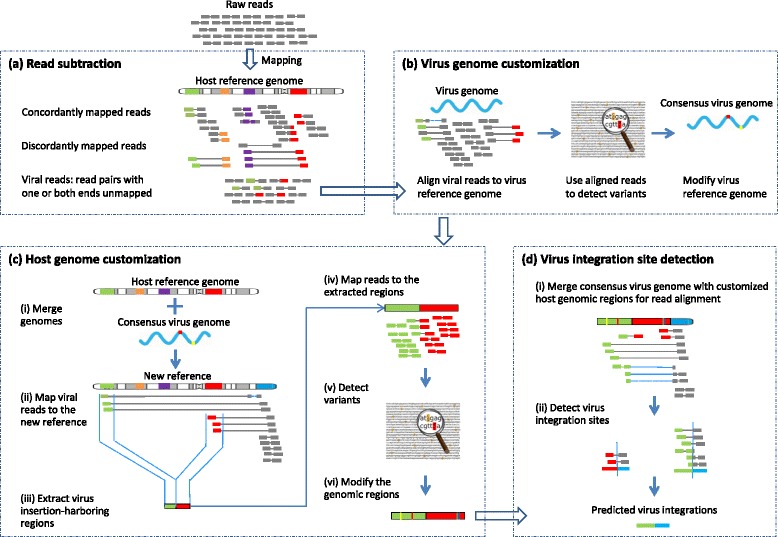
**Workflow of VERSE. (a)** Reads are aligned to a host reference genome. Unmapped reads and read pairs with one end unmapped are called viral reads. To differentiate mapped reads in the figure from unmapped ones, which remain grey throughout the pipeline, the color of mapped reads is changed from grey to the color of the host genomic region they are aligned to. **(b)** The viral reads are mapped to a virus reference genome. The high-quality consensus SNPs and indels detected from aligned reads are used to modify the virus reference genome. **(c)** The consensus virus genome created is concatenated to the host reference genome (designated as a separate pseudo-chromosome, *chrVirus*). Next, the viral reads are mapped to the resulting new reference. Then, inter-chromosomal structural variants (SVs) are detected from aligned reads. The SVs involving both the host genome and *chrVirus* are used to infer virus integration-harboring regions in the host genome. Finally, using the same procedure as in (b), the identified host genomic regions are customized. **(d)** The modified host genomic regions are concatenated with the consensus virus genome. The viral reads are mapped to this new reference for the detection of inter-chromosomal SVs. The breakpoints of the SVs that involve both the virus and host genomes, if there are any, are reported as virus integration sites. In the figure, vertical dotted lines represent virus integration breakpoints.

Figure 1 illustrates the VERSE pipeline, which overall follows a four-step procedure: (a) read subtraction, (b) virus genome customization, (c) host genome customization, and (d) virus integration site detection.

#### Read subtraction

The purpose of the read subtraction step is to collect viral reads, that is, the reads characteristic of the viruses infecting a host. In this step, VERSE uses the alignment tool Bowtie 2 [35] to map raw sequencing reads to the reference genome of the host species under study. Bowtie 2 is run in its sensitive end-to-end mode in order to achieve high alignment speed. Read pairs with one or both ends unmapped to the host genome are garnered. These reads are called viral reads for simplicity, although not all of them are related to the viruses. VERSE exploits primarily the viral reads in order to detect virus integrations.

#### Virus genome customization

In this step, VERSE utilizes ICORN [36], a tool for correcting errors in small genomes, to customize virus reference genomes. Specifically, VERSE runs ICORN to map viral reads to the virus reference genomes and to identify SNPs and indels from the mapped reads. Only high-quality consensus SNPs and indels are used to modify the virus reference genomes. ICORN compares the coverage of the mapped reads at each base before and after the modifications. The corrections that reduced the coverage are rejected. The whole process, from read alignment and variant detection to base correction, is run iteratively. Typically, six iterations suffice to correct majority errors in a small reference genome [36].

#### Host genome customization

Customization of large genomes is time-consuming. For instance, to use short reads to modify the UCSC hg19, it can take ICORN over a week to complete at the Vanderbilt Advanced Computing Center for Research and Education (ACCRE) [37]. To speed up VERSE, we extract genomic regions from host genomes that are likely to harbor virus integration sites. We run ICORN only on the identified regions.

Specifically, we first combine the reference genome of the host species under study with the consensus virus genome created in the previous step (designated as a separate pseudo-chromosome, *chrVirus*). Next, we use BWA [38] to align the viral reads to the resulting new reference. With the alignment file created, VERSE runs SVDetect [39], a software tool that uses anomalously mapped read pairs to localize genomic rearrangements, to call inter-chromosomal SVs that involve both the host genome and *chrVirus*. The host genomic regions that potentially harbor virus integration sites are then derived from the mapped positions of the reads of the SVs (Figure 1c(ii)). These genomic regions are typically <10,000 bp in length, significantly smaller than the host genome.

Next, VERSE designates each region characterized from the host genome as a separate pseudo-chromosome. After concatenating them together, VERSE recruits reads mapped to these regions from the Bowtie 2-aligned file created in step (a). Then, following the same procedure as step (b), VERSE runs ICORN to iteratively align reads to these pseudo-chromosomes, call SNPs and indels from read alignment, and then correct the reference with the called SNPs and indels.

The final outputs of this step are therefore customized host genomic regions, each of which corresponds to a potential virus integration event. By modifying only these small regions, we are able to reduce the computation time from over a week on a large reference genome to a few hours.

#### Virus integration detection

In this step, VERSE concatenates the host genomic regions recruited in the previous step with the consensus virus genome to create an analysis-ready reference genome. VERSE runs BWA to map the viral reads to this new reference and then utilizes CREST [40] to detect inter-chromosomal SVs. CREST is an algorithm that exploits soft-clipped reads, the reads with partial alignments to the reference genomes, for SV identification. The breakpoints of the SVs that involve both the virus and host genomes, if there are any, are then reported as virus integration sites.

#### Result classification and filtering

As demonstrated above, VERSE combines two complementary tools, SVDetect [39] and CREST [40], to customize reference genomes and detect virus integration sites. SVDetect uses spanning reads, that is, paired-end reads with one end mapped to the host genome and another aligned to the virus genome, to characterize virus integration loci. It is fast but not able to discern integration breakpoints accurately. In contrast to SVDetect, CREST utilizes soft-clipped reads, which are potentially split reads that harbor virus integration breakpoints within themselves. CREST is prone to miss true-positive loci due to the difficulty to map split reads. But it is able to determine virus integration sites at single-base resolution. By combining SVDetect and CREST, VERSE balances computational efficiency and detection accuracy.

To measure the confidence of a predicted position relative to the real virus integration site, and based on the output of CREST, VERSE categorizes a prediction into one of two classes: (a) high confidence - if there are sufficient soft-clipped reads to support an integration locus so that CREST is able to detect it; and (b) low confidence - CREST fails to detect it for the lack of high-quality soft-clipped reads.

For a high-confidence prediction, CREST’s output is used directly as a putative virus integration site. For a low-confidence one, however, VERSE predicts its position based on the soft-clipped reads that cover it. In particular, VERSE derives the boundaries of the region that potentially harbor the integration site from the output of SVDetect. Next, VERSE sorts the loci within the boundaries in the descending order of the number of soft-clipped reads that are aligned to them. The one covered with the most soft-clipped reads is then used as an estimate of the real integration locus.

To discard the possible false-positives, VERSE requires the distance between two adjacent low-confidence virus integration sites to be at least 10 bp. The drawback of the use of this stringent cutoff is that VERSE could mistakenly discard a real integration event if it is within 10 bp of another.

### Input of VERSE

The input of VERSE includes NGS reads (in FASTQ format) sequenced from a host, a reference host genome (in FASTA format), and a reference virus genome (FASTA format). The entire pipeline of VERSE, from the initial read subtraction step to virus integration detection to result classification and filtering, is fully automated. The output of each step is used automatically as the input for the next step of the pipeline.

VERSE provides an argument *sensitivity_level* to allow users to designate the number of iterations of reference genome customization. Because the majority of errors in a reference gnome can be corrected using one or two rounds of ICORN iteration, when evaluating VERSE on the human tumors and cell lines in the section below, we let *sensitivity_level* = 1 for simplicity and to save time. We encourage users to tune its value in their applications so as to adjust VERSE’s detection sensitivity.

Another input argument of VERSE is *flank_region_size*, which defines the size of the flanking regions upstream and downstream of a genomic region under study. In our experiments presented in the section below, *flank_region_size* was set to the default value of 4,000. This means VERSE will search both the upstream 4,000 bp and downstream 4,000 bp regions flanking a genomic segment predicted by SVDetect to harbor a candidate virus integration site. By allowing VERSE to examine the flanking regions, we reduce the chance to miss virus insertion sites therein.

As mentioned above, the source code of VERSE is publicly available, through the open source software package VirusFinder 2 [30]. As the core module of VirusFinder 2, VERSE is utilized by VirusFinder 2 to characterize virus integration loci (in its sensitive detection mode; see user’s manual at [30]).

## Results and discussion

In this section, we first evaluate VERSE’s capability to customize virus reference genomes. Then, we examine the effect of host genome customization and VERSE’s performance in identifying virus integration in human tumor genomes. The utility of VERSE is available through the VirusFinder 2 software.

### Effect of virus genome customization

Although the viruses infecting the human tumors and cancer cell lines collected by us were reported by earlier studies, their consensus sequences are unknown to investigators. In order to estimate the resemblance of the VERSE-created consensus virus genomes with the intra-host virus populations, we ran VERSE on the simulation data.

The mutated copy of the HPV-16 virus reference genome (GI:310698439) that was inserted in the simulation data harbors 75 SNPs and 9 indels. VERSE recruited the viral reads by aligning the raw simulated reads to the UCSC hg19 sequence. Then, with the HPV-16 reference genome as input, VERSE used the viral reads to customize the HPV-16 reference. After one round of ICORN iteration, 68 (91%) out of 75 SNPs and 6 (67%) out of 9 indels (relative to the assembly of short reads) were successfully characterized in the HPV-16 reference and corrected. This result demonstrates that VERSE could accurately identify the consensus mutations of the intra-host virus populations.

Next, we compared the consensus virus genome produced by VERSE with the initial virus sequence that was inserted into the simulation data. The identity of the resulting consensus virus genome with the initial sequence is 99.9%, in comparison with 99.1% between the HPV-16 virus reference and the initial inserted sequence. This result indicates that starting from a commonly used virus reference genome, VERSE is able to generate a consensus virus genome that better represents the virus populations within a host.

### Effect of host genome customization

To show how well VERSE customizes host genomes, we randomly chose a WGS sample, 26 T, from the 13 HCCs (Table S1 in Additional file 1). The average coverage of 26 T is 65.5×. The HBV virus fused into this tumor genome at chr18:107920 as reported in [7].

We ran VERSE on the sequencing data of 26 T. In total, 0.1 billion (4.1%) out of 2.4 billion reads were recruited as viral reads. In step (c) of the pipeline, VERSE used SVDetect to identify three genomic regions (Chr3:140567185-140575795, Chr6:33823990-33832089, and Chr18:102747-110847) as putative virus integration-harboring regions. It is easy to see that the real virus integration site, Chr18:107920, is located in the third region.

Next, VERSE ran ICORN to iteratively customize the three genomic regions. In each round of ICORN customization, short reads were mapped against the three regions and then consensus SNPs and indels were identified from these regions for reference corrections. The coverage of the mapped reads at each modified base before and after correction was compared and the correction that reduced the coverage was rejected. We performed 6 rounds of customization on this sample. Table 2 summarizes the total number of corrections accepted in the three regions after ICORN terminated. As expected, majority corrections (87%) occurred in the third region, which harbors the known virus integration site.

**Table 2:** **Total number of SNPs and indels corrected for tumor genome 26 T**

**Putative virus integration-harboring regions** ^**a**^	**SNPs**	**Insertions**	**Deletions**
Chr3:140,567,185-140,575,795	26	6	2
Chr6:33,823,990-33,832,089	0	0	0
Chr18:102,747-110,847	178	5	9

^a^These three genomic regions were derived from SVDetect’s output. The last region harbors a HBV integration site.

**Figure 2:**
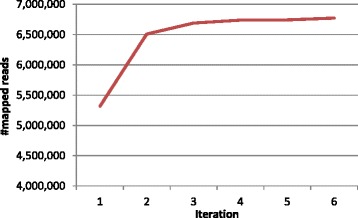
**The number of mapped reads as a function of ICORN iteration.** The total number of reads mapped to the three genomic regions (Chr3:140567185-140575795, Chr6:33823990-33832089, and Chr18:102747-110847) after each round of ICORN iteration. These three regions were derived from SVDetect’s output for HBV+ tumor sample 26 T (Table S1 in Additional file 1). The last region harbors a HBV integration site.

Figure 2 shows that read alignment was enhanced substantially after correcting SNPs and indels in the human reference genome. In particular, alignment improvement is more significant in the first two iterations, due to the reason that the majority of nucleotide corrections occurred therein.

With improved alignment, VERSE is able to recruit more reads (of higher mapping quality) to detect virus integration sites. An in-depth discussion of VERSE’s performance in detecting virus integration sites is the topic of the next section.

### Virus integration site detection

More than half of our samples used WGS, currently the most powerful sequencing technology. It provides the most comprehensive and unbiased characterization of genomic alterations (for example, gene fusions [41]) in genomes. Many discovery-based studies applied WGS technology to investigate the genome-wide associations between virus integration and tumor genomic instability [7-9]. One drawback of WGS is that, due to high sequencing cost, the sequencing coverage of WGS is typically between 30× and 60×, lower than that of other sequencing technologies, such as whole exome sequencing and targeted sequencing. Another challenge is that WGS requires intensive computational analysis, for which many existing tools are not capable.

In a previous study [28], WGS samples were used to compare the computational efficiency of three software tools: VirusSeq, ViralFusionSeq, and VirusFinder. That study, however, did not evaluate the capabilities of the three tools for detecting virus integration sites. With the lack of benchmark evaluation, the accuracy of virus integration site identification from the WGS data remains unclear to investigators. To the best of our knowledge, the results below present the first systematic evaluation of the sensitivity of virus integration detection in the WGS samples.

As demonstrated previously [28], several virus integration detection software, such as VirusSeq and ViralFusionSeq, require exceedingly high CPU use when analyzing WGS data. Considering the limited hardware resources, here we compare VERSE primarily against VirusFinder [28] on the WGS samples. VirusFinder is an efficient computational tool for analyzing NGS data. In our benchmark experiments, we ran VirusFinder in its *normal* mode (see user’s manual at [30]).

Table 3 summarizes our benchmark results on the human tumors and cancer cell lines. From the WGS samples, VERSE detected 16 (80%) out of 20 virus insertion events, compared with 13 (65%) detected by VirusFinder. Considering the difficulty in identifying virus integration sites, and in comparison with somatic single nucleotide variant detection, which has been intensely studied in the scientific communities and hence represents a more mature technology, VERSE performed reasonably well on this test data. Of note, the sensitivity of the state-of-the-art single nucleotide variant-calling tools is estimated to be only 81 to 86% [42].

**Table 3:** **The number of virus integration sites detected by VirusFinder, VirusSeq, and VERSE**

**Data type**	**Known integration sites**	**VERSE**	**VirusFinder** ^**a**^	**VirusSeq** ^**b**^
WGS	20	16	13	-
RNA-seq	11	9	8	7
TS	3	3	2	3
Total	34	28 (82%)	23 (68%)	-

^a^The version of VirusVinder used in our experiment is release 6/19/2014. ^b^The version of VirusSeq used in our experiment is the latest release (8/9/2013). RNA-seq, whole transciptome sequencing; TS, targeted sequencing; WGS, whole genome sequencing.

Additionally, Table 3 shows their comparative results on the RNA-seq and TS samples. Again, VERSE outperformed VirusFinder, characterizing successfully 12 (86%) out of 14 virus integration sites in these samples. Putting all these results together, the overall sensitivity of VERSE on our test data is 82% (28 out of 34), substantially higher than 68% by VirusFinder.

Table 3 also presents the number of virus integration sites detected by another tool, VirusSeq. VirusSeq is the first public software in this field for identifying virus integration sites in human tumor genomes. We downloaded the latest version of VirusSeq and ran it under its default setting on our test data. As indicated in Table 3, VirusSeq identified 10 (71%) out of 14 virus integration sites from the RNA-seq and TS samples, less than the 12 (86%) detected by VERSE (see the overlap of their detection results in Additional file 2).

Finally, it may be worth mentioning the computational efficiency of VERSE. With its well-designed pipeline, it takes VERSE on average <3 days (using 8 CPUs) to analyze a WGS sample on ACCRE (Table S3 in Additional file 1), in comparison with 14 days by ViralFusionSeq and >11 days by VirusSeq, as evaluated in [28]. This makes VERSE ideal for efficient analysis of large-scale sequencing data. VERSE’s speed and accuracy, together with its applicability to a wide array of NGS platforms (WGS, RNA-seq, and TS), will greatly benefit researchers in the field of virus sequencing studies.

## Conclusions

Pathogenic viruses are constant health threats across the globe. With the rapid advances in NGS technologies over the past several years and their widespread applications in clinical settings, there is an increasing interest in applying NGS to investigate the etiologic associations of viruses with diseases, especially human cancer. Accurate and comprehensive characterization of intra-host viruses would not only improve our understanding of host-pathogen interactions, molecular mechanisms of human diseases, and genome evolution, but also facilitate the development of successful antiviral treatments.

In this paper, we present VERSE, a novel approach that enhances virus integration detection through better read alignment. In particular, VERSE customizes both virus and host reference genomes to create personalized reference genomes, to which short reads align more easily. With improved alignment, VERSE is able to recruit more reads (of higher mapping quality) to detect virus integration sites. Using 19 human tumors and cancer cell lines as test data, we demonstrated that VERSE improved detection sensitivity substantially. VERSE has been implemented in our open source package VirusFinder 2 [30].

VERSE requires the presence of both spanning and soft-clipped reads to nominate a virus integration event. Comparing with other tools that focus either on spanning reads or on split reads, this is a stringent requirement. Though effective and fast, a potential drawback of this design is that it may miss real virus integration events if there is no supportive soft-clipped reads. Further improvement is needed in order not to miss real virus integration sites. Another limitation of VERSE is that ICORN, the tool used in VERSE for customizing reference genomes, cannot process single-end reads. This limits the applications of VERSE to paired-end sequencing data at present.

## Additional files

Additional file 1: Tables S1 to S3. Thirteen WGS samples and the validated HBV virus integration sites detected in them are described in Table S1. Four RNA-seq samples and the validated viral-human chimeric transcripts identified in them are provided in Table S2. Computation time of VERSE and VirusFinder on 10 whole genome sequencing samples is provided in Table S3. Additional file 2: Figure S1. Overlap of the detection results of VERSE, VirusFinder and VirusSeq on RNA-seq and targeted sequencing samples.

## Abbreviations

ACCRE: Advanced Computing Center for Research and Education
bp: base pair
HBV: hepatitis B virus
HCC: hepatocellular carcinoma
HIV: human immunodeficiency virus
HPV: human papillomavirus
indel: insertion and deletion
NGS: next generation sequencing
PCR: polymerase chain reaction
SNP: single nucleotide polymorphism
SV: structural variant
TS: targeted sequencing
WGS: whole genome sequencing
