# Supplementary figures and images for: VERSE: a novel approach to detect virus integration in host genomes through reference genome customization
Source: Genome Med. 2015 Jan 20;7(1):2. doi: 10.1186/s13073-015-0126-6 (PMC4333248; doi:10.1186/s13073-015-0126-6)

## Slide 1
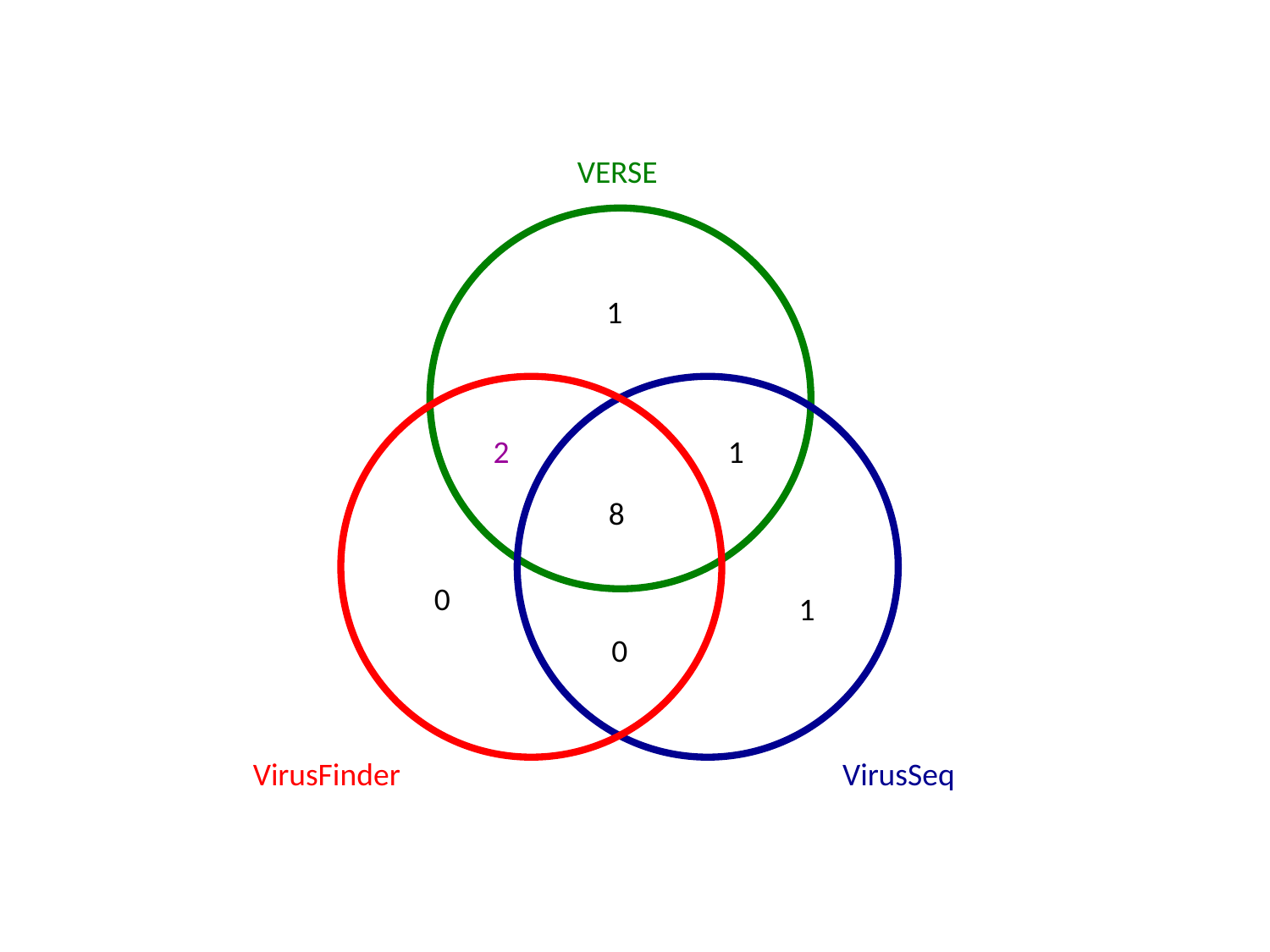

VERSE
1
2
1
8
0
1
0
VirusFinder
VirusSeq

Supplement: Additional file 2: Figure S1. — Overlap of the detection results of VERSE, VirusFinder and VirusSeq on RNA-seq and targeted sequencing samples. [file 13073_2015_126_MOESM2_ESM.ppt]
